# Course of the sFlt-1/PlGF ratio in fetal growth restriction and correlation with biometric measurements, feto-maternal Doppler parameters and time to delivery

**DOI:** 10.1007/s00404-021-06186-5

**Published:** 2021-08-25

**Authors:** A. Andrikos, D. Andrikos, B. Schmidt, C. Birdir, R. Kimmig, A. Gellhaus, A. Köninger

**Affiliations:** 1 Department of Obstetrics and Gynecology, University Hospital Essen, Essen, Germany; 2 Institute for Medical Informatics, Biometry and Epidemiology, University of Duisburg-Essen, Essen, Germany; 3 Department of Obstetrics and Gynecology, University Hospital Carl Gustav Carus, Dresden, Germany; 4 Department of Obstetrics and Gynecology, University Hospital Regensburg, Regensburg, Germany

**Keywords:** Fetal growth restriction, sFlt-1/PlGF, Soluble Fms-like tyrosine kinase 1, Placental growth factor, Time to delivery, Feto-maternal Doppler parameters

## Abstract

**Purpose:**

The study aimed to assess the course of the soluble Fms-like tyrosine kinase 1 (sFlt-1)/placental growth factor (PlGF) ratio in pregnant women with fetal growth restriction (FGR) and to evaluate potential associations between the sFlt-1/PlGF ratio and feto-maternal Doppler parameters, fetal biometric measurements and the time between study inclusion and birth (“time to delivery”).

**Methods:**

This was a retrospective longitudinal single center study including 52 FGR cases. The serum levels of sFlt-1 and PlGF were measured by using the BRAHMS Kryptor Compact PLUS. Fetal biometric and Doppler parameters, as well as the sFlt-1/PlGF ratio, were obtained both upon study inclusion and upon birth.

**Results:**

Various associations between the levels of the biomarkers in maternal blood upon study inclusion and upon birth and sonographic parameters were observed in FGR cases: umbilical artery (*p* < 0.01), uterine arteries (*p* < 0.01), ductus venosus (*p* < 0.05), cerebroplacental ratio (CPR) (*p* < 0.01), femur length (*p* < 0.01) and birth weight (*p* < 0.01). The higher the sFlt-1/PlGF ratio upon study inclusion, the shorter the “time to delivery” (*p* < 0.01). The multivariate regression analysis showed that the greater the daily percentage increase of the angiogenic markers, the shorter the “time to delivery” (*p* < 0.01).

**Conclusion:**

The fetal well-being, as measured by feto-maternal Doppler parameters such as CPR and the severity of the placental dysfunction, as measured by the urgency of birth and birth weight, is reflected by the level of the sFlt-1/PlGF ratio in the maternal serum. A rapid daily increase of the sFlt-1/PlGF ratio is significantly associated with the clinical progression of the disease.

## Introduction

The fetus suffering from fetal growth restriction (FGR) does not reach its genetically predetermined growth due to an underlying pathology [1]. Uteroplacental insufficiency, which leads to oxygen deficiency for the fetus, appears to be the most common cause of FGR [2]. FGR is associated with increased rates of perinatal morbidity and mortality [3] and therefore requires close monitoring. Depending on gestational age (GA) a distinction is also made between early-onset FGR (< 34th week of pregnancy) and late-onset FGR (≥ 34th week of pregnancy).

Preeclampsia (PE) and FGR are two entities of impaired placental function, therefore clinical and pathophysiological overlapping is common. The pathogenesis and etiology of placental dysfunction have not yet been fully elucidated, however, the imbalance between angiogenic and antiangiogenic factors is considered to play a critical role in its development [4]. The placenta produces several proangiogenic factors such as vascular endothelial growth factor (VEGF), placental growth factor (PlGF) and antiangiogenic factors, such as soluble Fms-like tyrosine kinase 1 (sFlt-1) [5]. The balance between these factors is crucial for the normal development of the pregnancy and if this balance is compromised, it may lead to endothelial dysfunction in the maternal vascular system [6].

Placental hypoxia and oxidative stress lead to excessive production of sFlt-1 [5] and thus to a reduction of the concentrations of VEGF and PlGF in the maternal vascular system [7]. The impaired angiogenic balance appears to be the result of pathological placentation, however, it does not always result in clinical manifestation of maternal disease [8] or in FGR. Previous studies [9–11] analyzing human sFlt-1 FGR mouse models have shown that increased levels of sFlt-1 affect the vascularization in the murine placenta and consequently impair placental function leading to FGR fetuses. These findings implement that an elevated sFlt-1/PlGF ratio may not only cause endothelial dysfunction in the maternal vascular system, but also can affect per se the normal development of the placenta. The sFlt-1/PlGF ratio is significantly increased before the clinical manifestation of PE appears [4] and therefore, sFlt-1/PlGF is used to predict the occurrence of a PE [12]. Furthermore, sFlt-1/PlGF levels correlate with the severity of PE [13].

A high sFlt-1/PlGF ratio is also associated with FGR [8]. However, an elevated sFlt-1/PlGF ratio indicates placental pathology but is not able to discriminate between PE or FGR. In contrast to PE, sFlt-1/PlGF levels are not routinely evaluated in the clinic as predictors for the course of FGR.

This study aimed to assess the course of the sFlt-1/PlGF ratio in pregnant women with FGR fetuses and to evaluate potential associations between the sFlt-1/PlGF ratio and feto-maternal Doppler parameters, fetal biometric measurements and the time between study inclusion and birth in FGR (“time to delivery”).

## Materials and methods

### Study population

This study is a retrospective longitudinal study. All patients were treated between 2015 and 2017 in the Department of Obstetrics and Gynecology at the University Hospital Essen, Germany. A total of 52 patients were consecutively enrolled in the study. The patients were referred to the hospital with suspected FGR without PE. The severity of FGR of the patients enrolled in the study was different, varying from patients that needed to be delivered shortly after the first examination to patients where the pregnancy could be prolonged for several weeks. Blood serum for sFlt-1/PlGF determination was collected from every patient with suspected FGR in our hospital. Previously described cut-off values of the sFlt-1/PlGF ratio > 85 to predict early-onset preeclampsia [12] were used as an inclusion criterion. Patients with PE and without FGR, fetal chromosomal anomalies and congenital infections were excluded from this study. Patients were included independently of gestational age.

The various parameters as well as the sFlt-1/PlGF ratio were obtained at the time of the first examination and were defined as “parameters upon study inclusion”. The same parameters were obtained before birth and were defined as “parameters upon delivery”. The patients suffered from different severity of placental insufficiency upon study inclusion and not all of the patients were directly hospitalised until the birth. The hospitalisation was indicated if birth was impending based on sonographic, clinic or cardiotocographic criteria. The first determination of the various parameters upon study inclusion (first examination) and the last determination of the same parameters before birth were used for the statistical analysis.

All sonographic parameters, except for the uterine artery Doppler studies, which were performed only upon study inclusion, were obtained both upon study inclusion and upon birth. FGR is defined as an estimated fetal weight < 10th percentile and/or non-percentile appropriate fetal growth during pregnancy (arrest of fetal growth or change in its rate in at least 2 measurements performed 3 weeks apart from each other) and pathological Doppler of the umbilical artery (pulsatility index (PI) > 95th percentile) or pathological Doppler of the uterine artery (PI > 95th percentile) or oligohydramnios [14]. PE is defined as the new onset of hypertension ≥ 140/90 mmHg on 2 separate occasions ≥ 4 h apart and significant proteinuria ≥ 300 mg/24 h [15]. Written consent of the patients to participate in the study was obtained. The study was approved by the local ethics committee of the University of Duisburg-Essen (No. 12-5212-BO).

### Sampling of blood serum

9 ml of blood was collected from each woman using a serum monovette (Sarstedt AG and Co.), stored at 4 °C and processed within 4 h to avoid blood cell lysis. Blood fractionation was performed by centrifugation at 10 min for 2500×*g*. Subsequently, 400 μl of the upper phase, containing blood serum, were carefully removed and used for the measurement of sFlt-1 and PlGF levels.

### Determination of sFlt-1 and PlGF

At least 8 µl blood from the serum monovette is required in order to measure the concentration of sFlt-1 (BRAHMS sFlt-1, No. 845.075) and at least 70 µl blood for the measurement of the concentration of PlGF (BRAHMS PlGF plus, No. 859.075). The protein levels of sFlt-1 and PlGF were measured by using the BRAHMS KRYPTOR compact PLUS machine based on TRACE® Technology according to the manufacturer protocol (time-resolved amplified cryptate emission) (BRAHMS Kryptor Compact, Thermo Fischer Scientific, BRAHMS GmbH, Hennigsdorf, Germany). The lowest detection limit for sFlt-1 was assessed as being 22 pg/ml and for PlGF 3.6 pg/ml. The upper detection limit for sFlt-1 was assessed as being 90,000 pg/ml and for PlGF 7000 pg/ml. The functional assay sensitivity, detected by inter-assay precision corresponding to a 20% coefficient of variability (CV), has been calculated as being lower than 29 pg/mL for sFlt-1 and 6.7 pg/mL for PlGF.

### Ultrasound scan

The transabdominal ultrasound was performed using three different ultrasound devices: Voluson S8 (GE Healthcare, Chicago, Illinois), Voluson E8 (GE Healthcare, Chicago, Illinois) and Phillips iU22 (Phillips, Seattle, Washington). The ultrasound was carried out by qualified observers upon routine conditions and guidelines. Second and third trimester biometry was performed by measuring the abdominal circumference (AC), the biparietal diameter (BPD), head circumference (HC) and the femur length (FL). The fetal weight and the weight percentile were calculated using the Hadlock curves [16]. The following Doppler parameters were measured: PI of the uterine arteries (UtA), PI of the umbilical artery (UA), PI of the middle cerebral artery (MCA), PI of the ductus venosus (DV) and the cerebroplacental ratio (CPR) as the ratio between MCA PI and UA PI.

### Birth percentiles

Voigt’s percentile curves were used in this study to determine the birth weight percentiles [17].

### Analysis of sFlt-1/PlGF-ratio dynamics

The “time to delivery” was defined as the time in days between the first determination of the sFlt-1/PlGF ratio upon study inclusion and the last determination of the sFlt-1/PlGF ratio before birth. The difference between the levels of the angiogenic markers upon birth and upon study inclusion was measured and the absolute increase of the ratio was calculated. After dividing the difference with the “time to delivery”, the daily increase of the sFlt-1/PlGF ratio was obtained in absolute terms. The total percentage increase of the ratio was calculated as follows: the difference (increase) between the sFlt-1/PlGF ratio upon study inclusion and upon birth was measured. This increase was then divided by the original value × 100 (sFlt-1/PlGF upon study inclusion). By dividing the total percentage change by the period of time in days between study inclusion and birth (“time to delivery”), the daily percentage increase of the ratio was calculated.

### Criteria for delivery of the FGR fetus

Table 1 shows the in house guidelines of our department upon which the delivery was indicated in FGR cases depending on the GA. The in house guidelines were based on the following publications: [18, 19] (Table 1).

**Table 1:** Criteria for delivery of the FGR fetus depending on the GA [18, 19]

GA	Criteria for delivery
≤ 28 + 0 weeks	Reversed DV a-wave **and** UA reversed EDV **and** deepest vertical pocket of amniotic fluid < 2 cm **and** no fetal movements **or** reversed DV a-wave **and** pathological CTG (deceleration pattern)
28 + 0 – 30 + 6 weeks	Reversed DV a-wave **or** UA reversed EDV **and** deepest vertical pocket of amniotic fluid < 2 cm **and** no fetal movements
31 + 0 – 33 + 6 weeks	Absent UA EDV **or** absent DV a-wave **or** deepest vertical pocket of amniotic fluid < 2 cm **and** no fetal movements
≥ 34 + 0 weeks	DV PI > 95th percentile **or** UA PI > 95th percentile **or** MCA PI < 5th percentile **or** CPR < 5th percentile **or** amniotic fluid index < 5th percentile

*DV* ductus venosus, *UA* umbilical artery, *EDV* end diastolic velocity, *CTG* cardiotocography, *MCA* middle cerebral artery, *CPR* cerebroplacental ratio

### Statistical analysis

Statistical analyses were carried out using the statistical program SPSS (version 11.5) (SPSS Inc., Chicago, IL, USA). The descriptive data were presented using the mean, median, standard deviation (SD) and interquartile range (IQR). Spearman’s rank correlation test was used to examine whether the sFlt-1/PlGF ratio was correlated to the Doppler parameters, biometric measurements and the “time to delivery”, depending on normality checked with the Kolmogorov–Smirnov test and the Shapiro–Wilk test. The various parameters upon study inclusion and birth were compared using a Wilcoxon-rank sum test. Bivariate and multivariate linear regression models were applied on sFlt-1/PlGF and biometrical as well as Doppler parameters and on “time to delivery”, including all parameters that were significantly correlated at the univariate level.

## Results

The result part is divided in three sections:

1. sFlt-1/PlGF ratio und its association with sonographic parameters
2. sFlt-1/PlGF ratio and its association with the “time to delivery”
3. Course of sFlt-1/PlGF ratio in FGR patients with and without PE

### Study cohort

Patient characteristics, clinical and sonographic parameters are shown in Table 2. All patients presented with an isolated FGR, whereas 10 of those patients developed accompanying PE during the course of treatment. The pregnancy week upon study inclusion ranged from the 19th to the 39th week of pregnancy and was not considered as an inclusion criterion in this particular study. The mean gestational age (GA) in weeks after the last menstrual period (or early pregnancy scan) at the time of the first examination was 30 weeks (SD ± 5.3) and upon birth 32.7 weeks (SD ± 5), the mean maternal age was 29.8 (SD ± 6.4) years and the majority of the patients were primiparae (78.9%). The mean birth weight in our population was 1610 g (± 722) and the mean birth weight in percentiles was 7 (SD ± 7) (Table 2). The median difference between the last sFlt-1/PlGF determination and birth was 3 (1–7.25) days (Table 3). The sFlt-1/PlGF ratio upon study inclusion and upon birth and the parameters determined to describe the sFlt-1/PlGF ratio dynamics are shown in Table 3. The birth of the majority of the patients was indicated following the criteria depending on the GA showed in Table 1 (*N* = 42). For some of the patients the birth was indicated based on maternal conditions and PE (*N* = 8) and for 2 of the patients the birth was indicated due to abruptio placentae.

**Table 2:** Patient characteristics: clinical and sonographic parameters upon study inclusion and at the “time of delivery” in mean (± SD) and median (IQR)

Parameter	Upon study inclusion	Upon birth	*p*-value
Pregnancy BMI (kg/m^2^)	25.6 (± 4.52), 25.45 (22.47–28.10)	–	–
Maternal age	29.8 (± 6.4), 28 (24–35)	–	–
Gravidity	1.62 (± 1.27), 1 (1–2)	–	–
Systolic arterial blood pressure (mmHg)	127 (± 16), 129 (118–140)	131 (± 16), 130 (118–143)	0.06
Diastolic arterial blood pressure (mmHg)	77 (± 15), 77 (67–85)	78 (± 13), 77 (70–89)	0.50
PI right UtA,	1.40 (± 0.89), 1.29 (0.76–1.74)	–	–
PI left UtA,	1.43 (± 0.58), 1.42 (0.93–1.88)	–	–
PI UA	1.41 (± 0,56), 1.20 (1.03–1.78)	1.74 (± 1.32), 1.34 (1.06–1.89)	0.01
PI MCA	1.75 (± 0.41), 1.69 (1.52–2.07)	1.51 (± 0.34), 1.48 (1.23–1.74)	< 0.001
PI DV	0.69 (± 0.32), 0.61 (0.45–0.88)	0.72 (± 0.39), 0.61 (0.40–0.84)	0.91
CPR	1.47 (± 0.62), 1.52 (1.01–1.73)	1.25 (± 0.48), 1.16 (0.89–1.55)	< 0.001
BPD (percentiles)	15 (± 21), 4 (0.5–22)	10 (± 16), 4 (0.3–13)	0.008
AC (percentiles)	9 (± 11), 6 (1–15)	6 (± 8), 3 (0.4–9)	0.002
FL (percentiles)	14 (± 20), 4 (0.5–19)	11 (± 16), 4 (0.1–13)	0.001
Birth weight (g)	–	1610 (± 722), 1660 (1100–2173)	–
Birth height (cm)	–	41 (± 7), 43 (39–46)	–
Birth weight (percentiles)	–	7 (± 7), 5 (2–9)	–
Birth height (percentiles)	–	12 (± 12), 7 (4–17)	–

*SD* standard deviation, *IQR* interquartile range, *sFlt-1* soluble Fms-like tyrosine kinase 1, *PlGF* placental growth factor, *BMI* body mass index, *mmHg* millimeter of mercury, *GA* gestational age, *PI* pulsatility index, *UtA* uterine artery, *UA* umbilical artery, *MCA* middle cerebral artery, *DV* ductus venosus, *CPR* cerebroplacental ratio, *BPD* biparietal diameter, *AC* abdominal circumference, *FL* femur length, *g* grams, *cm* centimeters

**Table 3:** Parameters of the sFlt-1/PlGF ratio course

Parameter	Mean (± SD)	Median (IQR)
GA upon study inclusion in weeks after the last menstrual period	30 (± 5.3)	31.6 (26.7–34.1)
GA upon birth in weeks after the last menstrual period	32.7 (± 5)	34.1 (30.1–36.9)
“Time to delivery” (days)	19 (± 14)	16 (7–30)
Difference between the last sFlt-1/PlGF determination and birth in days	–	3 (1–7.25)
sFlt-1/PlGF ratio upon study inclusion	448 (± 746)	269 (96–545)
sFlt-1/PlGF ratio upon birth	590 (± 780)	394 (140–738)
Absolute increase of the sFlt-1/PlGF ratio	142 (± 296)	32 (0–159)
Absolute increase of the sFlt-1/PlGF ratio per day	7 (± 17)	1 (0–8)
Total percentage increase of the sFlt-1/PlGF ratio	208 (± 658)	9 (0–169)
Percentage increase of the sFlt-1/PlGF ratio per day	7 (± 20)	0.9 (0–6)

*SD* standard deviation, *IQR* interquartile range, *sFlt-1* soluble Fms-like tyrosine kinase 1, *PlGF* placental growth factor, *GA* gestational age

### Correlation between sFlt-1/PlGF ratio and sonographic parameters

Sonographic parameters were compared at the time of study inclusion and birth. The following parameters differed significantly between study inclusion and birth: AC (*p* = 0.002), BPD (*p* = 0.008), FL (*p* = 0.001), PI UA (*p* = 0.01), PI MCA (*p* < 0.001), CPR (*p* < 0.001). No differences were shown between the PI of the DV (*p* = 0.91), the systolic (*p* = 0.06) and diastolic arterial blood pressure (*p* = 0.50) upon study inclusion and birth (Table 2).

Various associations between the levels of the biomarkers in maternal blood and sonographic parameters were observed (Table 4). SFlt-1/PlGF upon study inclusion correlated significantly negatively with the AC (*p* = 0.03), the FL (*p* < 0.01), the PI of the MCA (*p* < 0.01), the CPR (*p* < 0.01) and positively with the PI of the right UtA (*p* = 0.03), the PI of the left UtA (*p* < 0.01) the PI of the UA (*p* < 0.01) and the PI of the DV (*p* = 0.01) (Table 4). Sflt-1/PlGF upon birth correlated significantly negatively with the FL (*p* < 0.01), the birth weight (*p* < 0.01), the birth height (*p* = 0.04), the CPR (*p* < 0.01) and positively with the PI of the UA (*p* < 0.01) and the PI of the DV (*p* = 0.02). The results are summarized in Table 4.

**Table 4:** Correlation analysis using the Spearman’s rank correlation test between various parameters and the sFlt-1/PlGF ratio upon study inclusion and birth

Parameter	sFlt-1 upon study inclusion		sFlt-1 upon birth	
	Correlation coefficient	*p*-value	Correlation coefficient	*p*-value
BPD	− 0.22	0.13	− 0.19	0.23
HC	0.04	0.76	0.18	0.26
AC	− 0.30	0.03	− 0.25	0.10
FL	− 0.40	< 0.01	− 0.44	< 0.01
PI right UtA	0.32	0.03	–	–
PI left UtA	0.43	< 0.01	–	–
PI UA	0.46	< 0.01	0.46	< 0.01
PI MCA	− 0.45	< 0.01	− 0.10	0.54
PI DV	0.42	0.01	0.40	0.02
CPR	− 0.54	< 0.01	− 0.44	< 0.01
Birth weight	–	–	− 0.47	< 0.01
Birth height	–	–	− 0.31	0.04

*sFlt-1* soluble Fms-like tyrosine kinase 1, *PlGF* placental growth factor, *BPD* biparietal diameter, *HC* head circumference, *AC* abdominal circumference, *FL* femur length, *PI* pulsatility index, *UtA* uterine artery, *UA* umbilical artery, *MCA* middle cerebral artery, *DV* ductus venosus, *CPR* cerebroplacental ratio

To clarify possible associations between independent and dependent variables, bivariate and multivariate linear regression models were applied on sFlt-1/PlGF ratio upon study inclusion and birth, including all parameters that were significantly correlated with the angiogenic marker at the univariate level. The multivariate linear regression analysis did not show any significant correlations (data not shown).

### Correlation between sFlt-1/PlGF and the “time to delivery”

A correlation analysis was also performed between the “time to delivery” given in days and sFlt-1/PlGF ratio upon study inclusion and birth and the several dynamic parameters characterizing the course of sFlt-1/PlGF increase. Associations were shown between the “time to delivery” and the sFlt-1/PlGF ratio upon study inclusion (*p* < 0.01), the GA upon study inclusion in weeks (*p* < 0.01) and with all parameters of increase of the sFlt-1/PlGF ratio in absolute and percentage terms (*p* < 0.01). The results are shown in Table 5.

**Table 5:** Correlation analysis using the Spearman’s rank correlation test between the “time to delivery” and parameters characterizing the course of the sFlt-1/PlGF ratio

Parameters of the course of sFlt-1/PlGF ratio	“Time to delivery” Correlation coefficient *p*-value
sFlt-1/PlGF ratio upon study inclusion	− 0.40 < 0.01
sFlt-1/PlGF ratio upon birth	− 0.11 0.43
GA upon study inclusion in weeks	− 0.39 < 0.01
GA upon birth in weeks	0.01 0.97
Total absolute increase of sFlt-1/PlGF ratio	0.55 < 0.01
Daily absolute increase of sFlt-1/PlGF ratio	0.37 < 0.01
Total percentage increase of sFlt-1/PlGF ratio	0.60 < 0.01
Daily percentage increase of sFlt-1/PlGF ratio	0.49 < 0.01

*sFlt-1* soluble Fms-like tyrosine kinase 1, *PlGF* placental growth factor, *GA* gestational age

Bivariate linear regression models were applied on “time to delivery” including all parameters of increase of the sFlt-1/PlGF ratio in absolute and percentage terms. Significant correlations were observed between the “time to delivery” and the total absolute, the total percentage and the daily percentage increase of sFlt-1/PlGF ratio (*p* = 0.01, *p* = 0.02 and *p* = 0.03, respectively) (data not shown).

Multivariate linear regression models were subsequently applied on “time to delivery” including all parameters of increase of the sFlt-1/PlGF ratio that were significantly correlated with the “time to delivery” at the univariate level (Table 6). The multivariate linear regression analysis showed a negative correlation between the daily percentage increase of the sFlt-1/PlGF ratio and the “time to delivery” (*p* < 0.01) (Table 6). However, a positive correlation between the total percentage increase and the total absolute increase of the sFlt-1/PlGF ratio and the “time to delivery” was detected (Table 6).

**Table 6:** Results of the multivariate linear regression analysis of selected variables and the “time to delivery”

Regression parameter	ß estimate	*p*-value
Total absolute increase of sFlt-1/PlGF ratio	0.40	0.02
Total percentage increase of sFlt-1/PlGF ratio	2.26	< 0.001
Daily percentage increase of sFlt-1/PlGF ratio	− 2.13	< 0.01

*sFlt-1* soluble Fms-like tyrosine kinase 1, *PlGF* placental growth factor

### Comparison of patients with and without PE

The patients with isolated FGR presented a mean daily percentage increase of the sFlt-1/PlGF ratio of 5.77% (SD ± 11.4), whereas if PE was also present, a greater mean daily percentage increase of the sFlt-1/PlGF ratio (14.2% (SD ± 42.5)) was observed. However, the median daily percentage increase of the sFlt-1/PlGF ratio in the patients with isolated FGR was 1.23% (0–7.42), higher compared to the patients with accompanying PE 0.4% (0–3.4). Moreover, the mean sFlt-1/PlGF ratio upon study inclusion and the mean “time to delivery” did not show any significant differences between the patients with isolated FGR compared to the patients with accompanying PE (451 ± 806 vs 434 ± 439 and 18.9 ± 13.7 vs 18.2 ± 15.1, respectively). Furthermore, the mean sFlt-1/PlGF ratio upon birth was slightly but not significantly higher in the patients with isolated FGR compared to patients with accompanying PE (599 ± 847 vs 552 ± 425).

## Discussion

To our knowledge, this is the first study evaluating the respective course levels of sFlt-1/PlGF ratio in patients with FGR and correlating it with feto-maternal Doppler parameters, biometric measurements and the “time to delivery”. Various associations between the levels of the biomarkers in maternal serum and sonographic parameters were noticed in FGR cases. The course of the sFlt-1/PlGF ratio was associated with the “time to delivery” and the severity of the placental insufficiency was reflected by the levels of the angiogenic markers underscoring the importance of these biomarkers in pregnancies with placental dysfunction.

## sFlt-1/PlGF ratio and the “time to delivery”

The main outcome of this study is the negative correlation between the “time to delivery” and the daily percentage increase of sFlt-1/PlGF: the higher the daily percentage increase of the angiogenic markers, the more urgent is the birth, shown by a short “time to delivery”. A positive correlation between the total absolute increase and the total percentage increase of the sFlt-1/PlGF ratio and the “time to delivery” was seen, since patients who were admitted with rather lower levels of the sFlt-1/PlGF ratio and therefore were not delivered immediately, showed a greater increase of the ratio during ongoing pregnancy course.

Studies using larger cohorts conducted in patients with PE have shown similar results concerning a high sFlt-1/PlGF ratio and the necessity to deliver soon [7]. Therefore, it seems that the level of the sFlt-1/PlGF ratio reflects the severity of the placental insufficiency, as also described in previous studies [20]. The median “time to delivery” in our study was 16 days (7–30), which is similar to previous studies performed in patients with FGR [21].

In summary, we conclude that the higher the sFlt-1/PlGF ratio upon study inclusion, the more severe the placental dysfunction. Patients who were admitted with an extremely high sFlt-1/PlGF ratio showed only a small total percentage and absolute increase, since they started at a very high level. A longitudinal course of the angiogenic markers could not be observed in these patients due to the immediate birth. On the other hand, the patients who were admitted with a lower sFlt-1/PlGF ratio showed a greater increase of the biomarker because they had a lower sFlt-1/PlGF ratio upon study inclusion and the “time to delivery” was correspondingly longer. Thus, a positive correlation between the total percentage and absolute increase and the “time to delivery” was observable. On the contrary, the daily percentage increase correlated negatively with the “time to delivery” in both the bivariate and multivariate regression analysis, meaning that the steeper the daily percentage increase of the ratio, the faster these patients had to be delivered. This result appears plausible, since the daily percentage increase of the ratio reflects more objectively the progress of the placental dysfunction.

### sFlt-1/PlGF ratio and sonographic parameters

Sonographic determined AC and FL showed a strong correlation with the sFlt-1/PlGF ratio in our study. The AC is considered to be the best sonographic parameter for the diagnosis of FGR [22]. The insufficient blood flow to the gastrointestinal tract and to the liver leads to a small AC. A short FL is also an adaptive response to the “brain-sparing-effect” observed in cases of placental insufficiency [23]. The short femur has been associated with FGR in various previous studies [24, 25]. The BPD and the HC in FGR due to placental dysfunction is initially not affected by the redistribution of the blood flow to the vital organs [26], therefore the BPD and the HC could not be significantly correlated with the sFlt-1/PlGF ratio. Furthermore, the angiogenic markers upon birth were associated with the birth weight and height and therefore reflected the severity of the placental dysfunction in our study.

The progress of placental dysfunction in patients with FGR is characterized by the deterioration of the Doppler parameters and flattening of the growth curve, as clearly demonstrated by our data. An elevated sFlt-1/PlGF ratio has been associated by Navaratnam et al. with pathological Doppler indices of the UtA [27]. However, our study demonstrated that an increased sFlt-1/PlGF ratio is not only related to the maternal but also to the fetal Doppler parameters and consequently to the fetal well-being. The CPR has been associated in several studies with the perinatal outcome [28], in both early-onset FGR [29] und late-onset FGR [30]. Lobmaier et al. [31] showed that abnormal levels of PlGF in women with FGR in the third trimester of the pregnancy are associated with an adverse perinatal outcome. The same study demonstrated that Doppler parameters and angiogenic factors can predict adverse perinatal outcomes with similar performance. The strong association between the angiogenic markers and the CPR in our study and the currently available data indicate that the CPR could be classified as the Doppler parameter which correlates the most with the antiangiogenic expression profile of the placenta. Nevertheless, the isolated increase of the sFlt-1/PlGF ratio cannot currently be used in order to reach clinical decisions based exclusively on this increase. The delivery in patients with FGR should further be based on the GA and certain sonographic and cardiotocographic criteria [18, 19]. According to our study, the level of the sFlt-1/PlGF ratio could be used as an additional marker next to the Doppler parameters which may be helpful in order to assess the fetal well-being of FGR fetuses.

However, the multivariate linear regression analysis with the sFlt-1/PlGF ratio as dependent variable and the various sonographic and clinic parameters as independent variables did not show any significant correlations. The examined sonographic and clinic parameters represent different aspects of the placental dysfunction. Therefore, the sFlt-1/PlGF ratio can be assessed as a surrogate parameter for the diverse variables in our study but cannot be predicted based on solely one or more sonographic or clinic parameters.

### Subgroup of patients with additional PE

Although patients which developed PE showed similar sFlt-1/PlGF ratio at birth and similar “time to delivery” compared to FGR patients without additional PE, the mean daily percentage increase of the sFlt-1/PlGF ratio was higher in these cases, in accordance with former studies [32]. However, the median daily percentage increase of the ratio was higher in patients with isolated FGR. This result may depend on the low number of PE cases in our study. This small group of diagnosed PE patients could be due to the fact that since 2019 a new definition of preeclampsia has been included in the guidelines. Here hypertension and FGR are sufficient for the diagnosis of PE which may change the results. Given the fact that the values of the sFlt-1/PlGF ratio, the course of the biomarkers and the “time to delivery” did not show any significant differences between the patients with isolated FGR and those with accompanying PE and the fact that only 10 of the 52 patients (19.2%) developed PE in the course of treatment, we did not perform a subgroup analysis in this study. We conclude, that the observation of the daily changes of sFlt-1/PlGF contribute strongly to the severity of disease and the necessity for an urgent birth.

All the patients that were hospitalised in our institution received 40 mg low molecular weight heparin subcutaneous for deep vein thrombosis prophylaxis. Some studies [33, 34] suggest that the sFlt-1 levels in serum can be increased shortly after heparin administration. However, both studies [33, 34] showed that the sFlt-1 levels return to baseline in 10–12 h after heparin administration. Given the fact that our patients received heparin at 8 pm and the blood for the measurement of the sFlt-1/PlGF ratio was collected at the next morning, not earlier than 8am, suggest that the levels of the sFlt-1/PlGF ratio measured in our study were not affected or increased from heparin administration.

We acknowledge some limitations of the study. The small number of patients, the heterogeneity of the patient population and the different GA at the time of the first determination of the sFlt-1/PlGF ratio in our study are important factors that limit our ability to accurately predict the “time to delivery”. The role of the sFlt-1/PlGF ratio in predicting the “time to delivery” and the perinatal outcome in patients with placental dysfunction remains still limited, as also shown by other authors [35]. The sFlt-1/PlGF ratio should be obtained in further prospective studies at defined GA from the beginning of the second trimester to reexamine the associations between the course of the angiogenic markers and the “time to delivery”. To what extent a predefined level of the ratio in patients with FGR could lead to immediate birth should also be the subject of subsequent studies.

## Conclusion

The fetal well-being, as measured by feto-maternal Doppler parameters such as CPR and the severity of the placental dysfunction, as measured by the urgency of the birth and the birth weight, were reflected by the level of the sFlt-1/PlGF ratio in the maternal serum. A rapid daily percentage increase of the sFlt-1/PlGF ratio is significantly associated with the clinical progression of the disease.

## Data Availability

All data are available on request.

## References

[1] Wollmann HA (1998). Intrauterine growth restriction: definition and etiology. Horm Res.

[2] Berg C, Gembruch U, Geipel A, Rath W, Gembruch U, Schmidt S (2010). Intrauterine Wachstumsrestriktion. Geburtshilfe und Perinatalmedizin.

[3] Sheridan C (2005). Intrauterine growth restriction—diagnosis and management. Aust Fam Phys.

[4] Levine RJ, Maynard SE, Qian C, Lim KH, England LJ, Yu KF, Schisterman EF, Thadhani R, Sachs BP, Epstein FH, Sibai BM, Sukhatme VP, Karumanchi SA (2004). Circulating angiogenic factors and the risk of preeclampsia. N Engl J Med.

[5] Wang A, Rana S, Karumanchi SA (2009). Preeclampsia: the role of angiogenic factors in its pathogenesis. Physiology.

[6] Maynard SE, Min JY, Merchan J, Lim KH, Li J, Mondal S, Libermann TA, Morgan JP, Sellke FW, Stillman IE, Epstein FH, Sukhatme VP, Karumanchi SA (2003). Excess placental soluble fms-like tyrosine kinase 1 (sFlt1) may contribute to endothelial dysfunction, hypertension, and proteinuria in preeclampsia. J Clin Invest.

[7] Verlohren S, Herraiz I, Lapaire O, Schlembach D, Moertl M, Zeisler H, Calda P, Holzgreve W, Galindo A, Engels T, Denk B, Stepan H (2012). The sFlt-1/PlGF-ratio in different types of hypertensive pregnancy disorders and its prognostic potential in preeclamptic patients. Am J Obstet Gynecol.

[8] Herraiz I, Simón E, Gómez-Arriaga PI, Martínez-Moratalla JM, García-Burguillo A, López Jiménez EA, Galindo A (2015). Angiogenesis-related biomarkers (sFlt-1/PlGF) in the prediction and diagnosis of placental dysfunction: an approach for clinical integration. Int J Mol Sci.

[9] Kumasawa K, Ikawa M, Kidoya H, Hasuwa H, Saito-Fujita T, Morioka Y, Takakura N, Kimura T, Okabe M (2011). Pravastatin induces placental growth factor (PGF) and ameliorates preeclampsia in a mouse model. Proc Natl Acad Sci USA.

[10] Kühnel E, Kleff V, Stojanovska V, Kaiser S, Waldschütz R, Herse F, Plösch T, Winterhager E, Gellhaus A (2017). Placental-specific overexpression of sFlt-1 alters trophoblast differentiation and nutrient transporter expression in an FGR mouse model. J Cell Biochem.

[11] Vogtmann R, Kühnel E, Dicke N, Verkaik-Schakel RN, Plösch T, Schorle H, Stojanovska V, Herse F, Köninger A, Kimmig R, Winterhager E, Gellhaus A (2019). Human sFLT1 leads to severe changes in placental differentiation and vascularization in a transgenic hsFLT1/rtTA FGR mouse model. Front Endocrinol (Lausanne).

[12] Verlohren S, Herraiz I, Lapaire O, Schlembach D, Zeisler H, Calda P, Sabria J, Markfeld-Erol F, Galindo A, Schoofs K, Denk B, Stepan H (2014). New gestational phase-specific cutoff values for the use of the soluble fms-like tyrosine kinase-1/placental growth factor ratio as a diagnostic test for preeclampsia. Hypertension.

[13] Stepan H, Herraiz I, Schlembach D, Verlohren S, Brennecke S, Chantraine F, Klein E, Lapaire O, Llurba E, Ramoni A, Vatish M, Wertaschnigg D, Galindo A (2015). Implementation of the sFlt-1/PlGF ratio for prediction and diagnosis of pre-eclampsia in singleton pregnancy: implications for clinical practice. Ultrasound Obstet Gynecol.

[14] Kehl S, Dötsch J, Hecher K, Schlembach D, Schmitz D, Stepan H, Gembruch U (2016) Intrauterine growth restriction. Guideline of the German Society of Gynecology and Obstetrics (S2k-Level, AWMF Registry No. 015/080), 26–27 10.1055/s-0043-118908 PMC5784232 29375144

[15] Brown MA, Lindheimer MD, de Swiet M, Van Assche A, Moutquin JM (2001) The classification and diagnosis of the hypertensive disorders of pregnancy: statement from the International Society for the Study of Hypertension in Pregnancy (ISSHP). Hypertens Pregnancy. 20, IX–XIV 10.1081/PRG-100104165 12044323

[16] Hadlock FP, Harrist RB, Sharman RS, Deter RL, Park SK (1985). Estimation of fetal weight with the use of head, body, and femur measurements—a prospective study. Am J Obstet Gynecol.

[17] Voigt M, Fusch C, Olbertz D, Hartmann K, Rochow N, Renken C, Schneider KTM (2006) Analyse des Neugeborenenkollektivs der Bundesrepublik Deutschland. 12. Mitteilung: Vorstellung engmaschiger Perzentilwerte (-kurven) für die Körpermaße Neugeborener. Geburtshilfe Frauenheilk. 66(10), 956–970

[18] Baschat AA (2010). Fetal growth restriction—from observation to intervention. J Perinat Med.

[19] Figueras F, Gratacós E (2014). Update on the diagnosis and classification of fetal growth restriction and proposal of a staged-based management protocol. Fetal Diagn Ther.

[20] Herraiz I, Dröge LA, Gómez-Montes E, Henrich W, Galindo A, Verlohren S (2014). Characterization of the soluble fms-like tyrosine kinase-1 to placental growth factor ratio in pregnancies complicated by fetal growth restriction. Obstet Gynecol.

[21] Quezada MS, Rodríguez-Calvo J, Villalaín C, Gómez-Arriaga PI, Galindo A, Herraiz I (2019). sFlt-1/PlGF ratio and timing of delivery in early-onset fetal growth restriction with antegrade umbilical artery flow. Ultrasound Obstet Gynecol.

[22] Chang TC, Robson SC, Boys RJ, Spencer JA (1992). Prediction of the small for gestational age infant: which ultrasonic measurement is best?. Obstet Gynecol.

[23] Weisz B, David AL, Chitty L, Peebles D, Pandya P, Patel P, Rodeck CH (2008). Association of isolated short femur in the mid-trimester fetus with perinatal outcome. Ultrasound Obstet Gynecol.

[24] D'Ambrosio V, Vena F, Marchetti C, Di Mascio D, Perrone S, Boccherini C, Pizzuti A, Benedetti Panici P, Giancotti A (2019). Midtrimester isolated short femur and perinatal outcomes: a systematic review and meta-analysis. Acta Obstet Gynecol Scand.

[25] Vermeer N, Bekker MN (2013). Association of isolated short fetal femur with intrauterine growth restriction. Prenat Diagn.

[26] Rizzo G, Capponi A, Talone PE, Arduini D, Romanini C (1996). Doppler indices from inferior vena cava and ductus venosus in predicting pH and oxygen tension in umbilical blood at cordocentesis in growth-retarded fetuses. Ultrasound Obstet Gynecol.

[27] Navaratnam K, Abreu P, Jorgensen A, Alfirevic A, Alfirevic Z (2019). Evaluation of agreement of placental growth factor (PlGF) tests and the soluble FMS-like tyrosine kinase 1 (sFlt-1)/PlGF ratio, comparison of predictive accuracy for pre-eclampsia, and relation to uterine artery Doppler and response to aspirin. J Matern Fetal Neonatal Med.

[28] Bahado-Singh RO, Kovanci E, Jeffres A, Oz U, Deren O, Copel J, Mari G (1999). The Doppler cerebroplacental ratio and perinatal outcome in intrauterine growth restriction. Am J Obstet Gynecol.

[29] Conde-Agudelo A, Villar J, Kennedy SH, Papageorghiou AT (2018). Predictive accuracy of cerebroplacental ratio for adverse perinatal and neurodevelopmental outcomes in suspected fetal growth restriction: systematic review and meta-analysis. Ultrasound Obstet Gynecol.

[30] Hernandez-Andrade E, Serralde JA, Cruz-Martinez R (2012). Can anomalies of fetal brain circulation be useful in the management of growth restricted fetuses?. Prenat Diagn.

[31] Lobmaier SM, Figueras F, Mercade I, Perello M, Peguero A, Crovetto F, Ortiz JU, Crispi F, Gratacós E (2014). Angiogenic factors vs Doppler surveillance in the prediction of adverse outcome among late-pregnancy small-for-gestational-age fetuses. Ultrasound Obstet Gynecol.

[32] Herraiz I, Quezada MS, Rodriguez-Calvo J, Gómez-Montes E, Villalaín C, Galindo A (2018). Longitudinal change of sFlt-1/PlGF-ratio in singleton pregnancy with early-onset fetal growth restriction. Ultrasound Obstet Gynecol.

[33] Hagmann H, Bossung V, Belaidi AA, Fridman A, Karumanchi SA (2014). Low-molecular weight heparin increases circulating sFlt-1 levels and enhances urinary elimination. PLoS ONE.

[34] Searle J, Mockel M, Gwosc S, Datwyler SA, Qadri F, Albert GI, Holert F, Isbruch A, Klug L, Muller DN, Dechend R, Muller R, Vollert JO, Slagman A, Mueller C, Herse F (2011). Heparin strongly induces soluble fms-like tyrosine kinase 1 release in vivo and in vitro—brief report. Arterioscler Thromb Vasc Biol.

[35] Graupner O, Lobmaier SM, Ortiz JU, Karge A, Kuschel B (2018). sFlt-1/PlGF-ratio for the prediction of the time of delivery. Arch Gynecol Obstet.

